# Supplementary material for: Transcriptional Insights of Oxidative Stress and Extracellular Traps in Lung Tissues of Fatal COVID-19 Cases
Source: Int J Mol Sci. 2023 Jan 31;24(3):2646. doi: 10.3390/ijms24032646 (PMC9917045; doi:10.3390/ijms24032646)
Supplement: Supplementary file 1 [file ijms-24-02646-s001.zip › ijms-2158825-supplementary/ijms-2158825-supplementary.pdf]

(a)

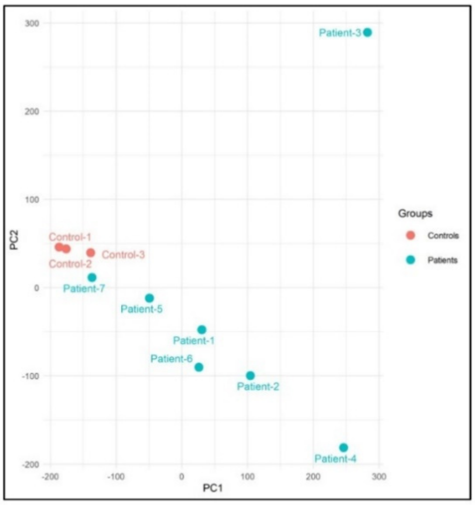

(b)

Signaling enrichment analysis

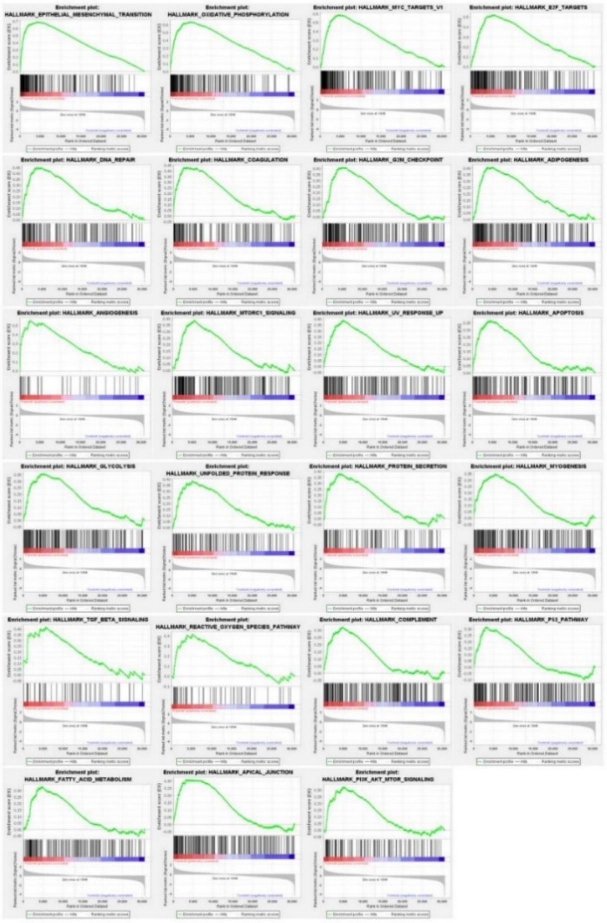

Figure S1: Principal-component analysis and signaling enrichment analysis.

(**a**) The scatterplot of principal-component analysis using normalized gene expression value of samples to indicate a distinct overlap of patients and controls. (**b**) Enriched signaling pathways with normalized *p* value < 0.001 in patients versus control individuals based on the hallmark gene set collection.

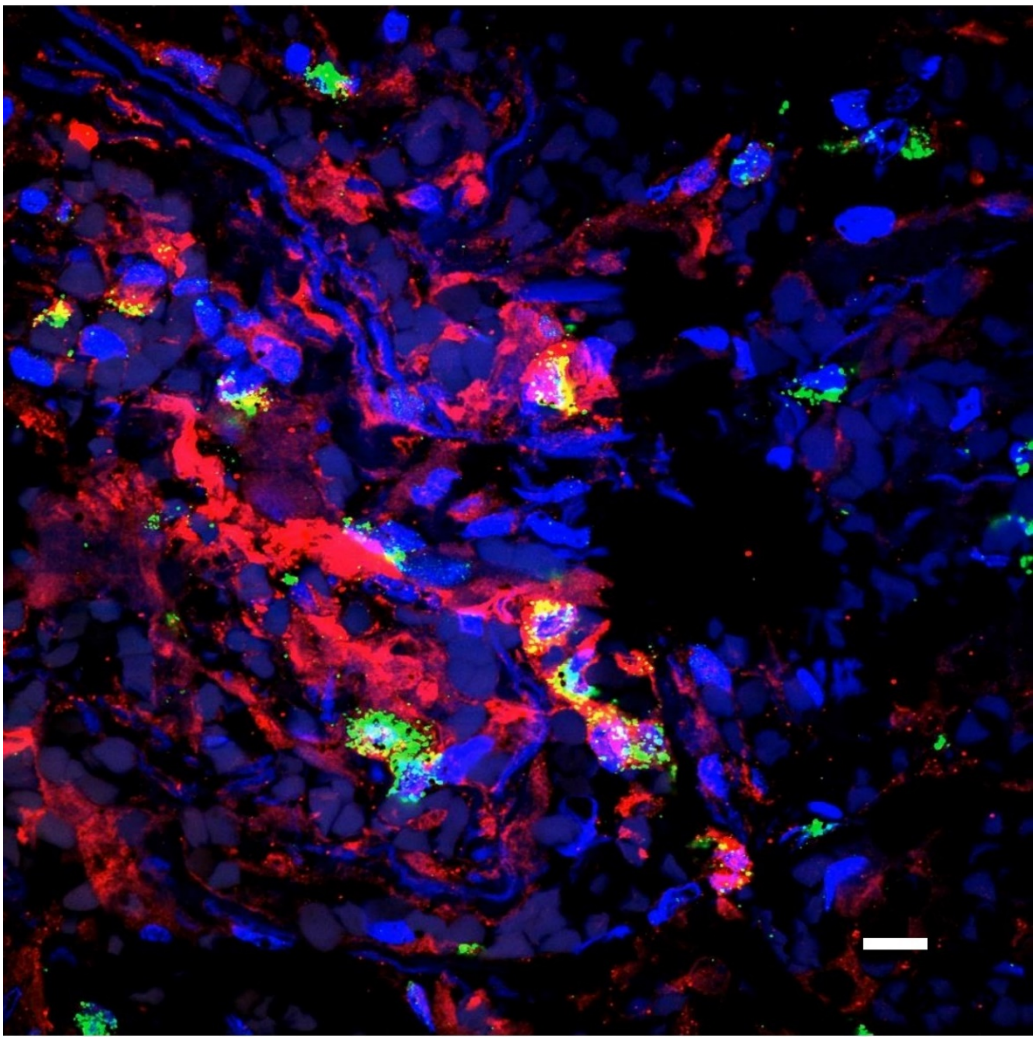

Supplementary Movie S1. NETs are co-localized with lipid oxidation marker 4-HNE.

Demonstration of extracellular DNA (Hoechst 33342, blue) and neutrophil granule protein (MPO, green) deposition together with the marker of lipid oxidation (4-HNE, red) under in vivo conditions (SARS-CoV-2 lung infection), with an interval of 0.1 µm, slices were taken throughout the z axis (z stacks) with 10 (left) and 15 (right) slices per stack in order to show extracellular DNA in their full extension. Scale bar, 10 µm, (n=3).

Table S1. List of differentially expressed genes between COVID-19 patients and healthy donors with corresponding logFCs and adjusted *p* values.
